# Account of Some Cases and Dissections

**Published:** 1808-11

**Authors:** Thomas Beddoes


### ACCOUNT OF SOME CASES AND DISSECTIONS.

By Thomas Beddoes, M. D.

THE uncertainty of medicine, notwithstanding partial improvements, which make some figure by themselves indeed, but dwindle on a collective estimate, is, I apprehend, too likely to continue. Nor do I see how it should cease, till careful observation of the sick be combined, beyond any example, past or present, with early and exact dissection of the dead. Our civil and military hospitals, considered with out complaisance, must be allowed to do the country credit —the military, from the excellent spirit of the medical officers, and the arrangements for the sick; the civil as demonstrating, upon the whole, that much charity of disposition pervades society. In the former, however, from numerous documents, just laid before parliament and the public, it is to be feared that intrigue can too easily contrive to domineer over tried merit; and the civil, as almost universally conducted, answer no one purpose more obviously

 than than giving unmerited eclat to a few medical men; such as by their connections can once for all secure a majority of votes among the subscribers. Of both the most enlarged view is melancholy enough. They are, if not quite barren, yet wretchedly unproductive of that sort of service, which, under a better system, they would yield to the public at large. For want either of voluntary combination, a thing scarce to be expected, or of (what is surely very feasible) regulation by authority, which, from regard to the general good, shall compel, as an absolute duty, the preservation and publication of every material fact, and thus make these humane establishments a thousand fold more serviceable to humanity, they furnish very equivocal testimony to the sense and spirit of a people, otherwise so enlightened and energetic as the English. Instructive phænomena must occur every where in turn; yet these are at present often confined to a petty circle, and soon totally sunk in oblivion. Must it not sometimes happen, that the connections and descendants of the observers themselves, much more, remote individuals, shall fall a sacrifice to disorders; for remedying which, the wider diffusion of these very observations would suggest the means? What is known in so narrow a sphere, for so short time, and to so little purpose, does by no means exhaust the topics of regret, supplied by our institutions for the sick. The opportunity for many discoveries is lost, because that ardour, which would spread into the obscurest corner, is not fully excited in these conspicuous stations of practice. Were it understood, how constantly inward diseases are the cause of perplexity and blunders, (a fact which, in regard to persons in the highest sphere of life, round whom the supposed brightest luminaries of physic were assembled, has repeatedly, of late, come to my certain knowledge) the moral of such a representation would be greatly strengthened. Nor should I be surprized to hear a warm-hearted spectator of mankind, if he were aware of our helplessness, and accustomed to discriminate between substance and shew, performance and profession, pronouncing that, *in a civilized state, physic ought not to be at all tolerated, or that much more effectual means than any in use ought to be taken, to insure discernment to the physician.*

Among disorders, where practice is unsettled, frequently therefore unsuccessful, and probably destructive, must be numbered, (notwithstanding some useful modern suggestions) those in which the head is affected, or a disturbance of the sensorial functions is accompanied by extreme debility.

lity. On this subject, well known discussions had rendered me particularly desirous of recurring to Nature herself. The occasion has offered itself oftener and more favourably than I could expect. In judging of the state of parts after death, it is, as in other instances, where any niceness of comparison is requisite. Upon impressions, made at long intervals, little reliance can be placed. He who examines a body once only in a quarter of a year, or once in a month, will be able to form but an insecure judgment, except in very palpable changes of structure. May I therefore be permitted to state that, besides long and frequent familiarity with the interior of animals, I was, by favour, allowed once to assist at as many as three recent human dissections in a day, where one or other of the parties present had observed the symptoms. I have repeatedly assisted at three in a week; the subjects commonly fresh, and a leisurely examination of the parts principally diseased, often following the general inspection. Though I may be exceeded in this advantage by a few, I yield to no one in the cultivation of an acquaintance with the most accurate descriptions and engravings. These instruct more or less as the party is prepared by knowledge of the objects; with which I have not neglected to compare, upon the spot, the the most esteemed plates. I have besides canvassed appearances at the moment, or afterwards, with observers from various climates. I hope, therefore, that the following Reports may deserve to be taken into account with others. In each article I give its peculiar circumstances, as the date of the dissection, &c. I relate the whole, not pretending at present to class facts, or to assign to particular symptoms particular alterations, though I constantly perceived appearances little connected with the fatal complaint. As no account, however, can put the reader on a footing with the spectator, some inferences shall hereafter be drawn, which observation alone could supply, lest any thing material should be wanting.

CASE I.

X. X. æt. 35, slender, puny, valetudinarian, mother of several children, recovering slowly after child-birth, then, and at other times, for near twenty years, subject to such cough and feverishness, as excited the apprehension of consumption, though there was no title to it from either parent; subject to sick head-ache; with dark hair, eyes, and skin.

I did not see this patient during the present illness; but after the first fortnight, was consulted by a medical friend, in the out-skirts of London, who nursed her from the 11th of May, 1808. For six months her health had appeared to improve, except that a few weeks ago she had had cough with feverishness, which had gone off.

May 2d, much fatigued by exercise in the meridian sun. 3d, was only sensible of having caught cold. 4th, pain of the left side of the chest; dyspnœa; short cough; leeches and blistering seemed to give relief. Soon after, the pain appeared to remove to the epigastrium and loins, and was accompained by fulness of abdomen, constant eructations, borborygmi, and constipation; fæces hard, scanty, dark. For this she had purgatives with calomel. Extreme debility now led to apprehend the accession of rapid and dangerous typhus.

Wednesday 11th, pulse 150. Thursday morning at 9, A. M. great prostration of strength; insensible, except roused; some muttering delirium; pulse 130, very irregular, frequently intermitting; "*not so feeble as I expected.*" Action of the heart very strong, as appeared from the touch and sound both. Tongue pale, very little incrusted; *no head-ache;* less pain and tension of abdomen, borborygmi; urine scanty, not high coloured; two or three stools a day, almost black, fœtid, with little substance. Short, frequent, loose cough; little pain remaining in the chest.

℞. Calomel gr. ii. Rhei. gr. v.; with mist. salinos; containing of infus. sennae t. ℥iii. every third or fourth hour.

For food gruel with a *very little* wine; which last, on some little amendment of appetite, and great cough during two nights, was withdrawn on the 14th. Afterwards acids were used with digitalis, mercurial inunction, and tepid ablution during the hot stage.

The hepatic action as indicated by bilious stools, and the violent beating of the heart, continued uninfluenced by any medicine. The febrile heats were much assuaged by ablution, but recurred with violence, and increased pulse from the 20th to the 27th. Abdomen soft, and free from pain, only a little distended by wind; stools; sleep absent, disturbed, or short; little delirium, as before.

27th. Slept from midnight till 5 A. M. composedly; sick, repeatedly vomited fresh green bile; stools ceased. Noon, oppression about stomach, rising in throat, dry retching. Stimulating glyster without effect. Now she began for the first time to complain of head-ache; but having having been accustomed to this with a bilious stomach, she supposed that the cause was now the same.—Evening, the head-ache suddenly increased; accession of delirium. Two physicians suppose a transition of morbid action from the liver to the brain with sudden effusion. They forbid bleeding and vomiting; advise digitalis, sinapisms, vesication. P. 120, not feeble. Coma towards morning; fluctuations in the heart and pulse. Afternoon, respiration laborious; pulse somewhat strong, heat considerable, (face rather florid, notwithstanding death had been hourly expected.) Six ounces of blood drawn. Pulse sensibly influenced, not much weakened, face paler; respiration slower. In a quarter of an hour pulse as vigorous as before, heat of the whole body *intense*, profuse perspiration following. Leeches applied to the temples. Blood from arm decidedly inflamed. Death about 3 A. M.; the pulse maintaining its strength till 1, and the perspiration profuse.

I know not whether the reader, who may first pause to deduce the internal state, will be disappointed by the following account; which I give, as received, in the words of a celebrated anatomist in London. I know not at what distance of time the dissection took place.

Stomach contained about one ounce of dirty greenish liquid. Its condition, and that of the bowels, natural. Colour and substance of the liver entirely free from disease; gall-bladder somewhat larger and more prominent than usual.

On the surface of the lungs, near the left branch of the trachea, appeared hard and irregular projections. *They felt as if scratching the hand, when it was passed behind the left lung.* They adhered to the pleura pulmonalis, were white, and distinctly osseous; seven or eight were counted. There were adhesions posteriorly to the pleura costalis. On dividing the substance, at the lower extremity, some congestion, and a small ulcer, (abscess?) were observed; right lung quite healthy. In each cavity, some bloody liquid; as also, and much more abundant, in the pericardium.

The general appearance of the heart was sound. But from the tricusped valve arose a large irregular indurated substance, of a whitish yellow colour, attached to the heart, and extending to the apex.

Is not this, with the profuse bloody effusion and constant excitement, proof of inflammation of the heart?

A conical polypus, stretching nearly through the ventricle, tricle, adhered to this matter, obviously of much more recent formation; the part nearest the valve having the appearance of lymph lately coagulated; the lower part of a deep red colour, like the ordinary crassamentum.

Beneath the *dura mater,* large serous effusion, appearing pearly from being seen through the tunica arachnoidea. The effusion extended on each side around the extremities, and below the base of the brain. In some parts, the fluid was of a yellowish colour, and some opacity, slightly resembling pus, or rather mucus, secreted in some undefined states of membranous disease. Rather more fluid than usual in the lateral ventricles. In other respects, the brain appeared natural.

### CASE II.

R. G. a sufficiently stout youth of 18.—The following is an abstract from the Journal of his case. I did not see him till after death. Violent pain of limbs, ascribed to a cold; complains of being chilly; skin dry and hot; p. 90, small; tongue slightly furred; bowels rather loose.—Emetic and twenty drops of tinct. of opium afterwards. Evening four stools.

18th. A restless night; delirium; tongue furred; skin hot; opiate; blister; enema, whence two stools.

19th. Bad night; sweats. Antimon. tart. gr. ii. aq. ℥vi. cochlear. ii. o. h.; sweat continued in the evening; complains of his chest; enema followed by two stools.

20th. Tolerable night; much the same otherwise. Antimon. tart. gr. i. and æther v. added to his former ℥vi. mixture; enema and two stools. Tinct. opii et vin. antimon. gtt. xxv. h. s.

21st. Good night, yet rather worse; tongue fouler; acid mixture with opiate and antimonial; enema. The day bad; delirium; two loose fœtid stools; tongue very foul; pulse 86, weak; "*disease putting on a putrid diathesis;*" skin hot and dry; camphorated opiate draught; lemons.

22d. In all respects worse; pulse 96, weak; irritable; loose stool; blister; camphor mixture with antimonial and æther; wine; blister again in the evening.

23d. Bad night; cold clammy sweat; died at half past four; opened next day.—So little deviating from a healthy state in any of the cavities, that after a full inspection of all the cavities, two surgeons accustomed to the opening bodies, asked each other, "*whether any thing had been seen here, which could account for the patient's death?*

The brain and its membranes shewed none of the appearances pearances which can be considered as inflammatory. The vascularity was even far below the standard of coloured engravings, professing to represent the healthy state: so was the number and size of the bloody points in the medullary substance. No effusion, remarkable hardness, or softness in the brain or cerebellum. Some slight adhesions in the thorax; heart rather flabby; the stomach was ruddier, especially towards the cardia, than in carnivorous animals killed in full health, or than in the human subject a very short time after death, in which, when there is no reason to suspect disease, the surface is quite pale.* But the present degree of ruddiness was indeed far below what we can venture to consider as high state of disease, and below what I have repeatedly seen in the same seat, and, from the analogy of inflammation elsewhere, suppose fatal.

### CASE III.

Another man, aged 28, had similar symptoms, only there was more delirium and bilious diarrhœa. The treatment appeared from the notes, (for I did not see the patient) very similar; the appearances in the head and stomach (dissection taking place the day of his death) very similar. The liver was hard and of a large size, but from the patient's habits, the alterations were probably not altogether recent, and can hardly be supposed fatal, if they were. The reader perceives the meagreness of this account, so does the writer. The scalpel often makes the most satisfactory disclosures. It is sometimes a miserable pathological tool; but it should be impressed upon us all, that similar sensorial movements may, or may not, be accompanied with given changes of other parts; that the present or a future age may bethink themselves of the means of distinguishing the difference, and even of reaching the source of the symptoms in the nervous matter itself.

### CASE IV.

B. J. aged 21, taken ill on the 16th of June, 1808, with slight

---

* Die Sept. 16to. e. g. ventriculum phthisici, calentem adhuc et prorsus sanum (nisi totum fortasse corpus sanguinis inopia laborasse) bihorio vix post mortem elapso, adstantibus Dnis *Felix*, *Marks*, *Lawrence*, et alio quodam chirurgo, lustravi. Tunicis integerrimis, extantibus pulchré rugis, color erat pallidus, subflavus seu potiús dilutè bruneus. Procul dubio ventriculos omnes, quos viderim typho extinctorum, si ad hunc contuleris, inflammatis adnumerare oportet.

slight pain in the head; did not apply for aid till the 18th of June; at that time the pain continued, attended with slight febrile symptoms; p. 90; nausea with a good deal of cough; free expectoration of mucus; not any pain about the thorax. He referred the pain in the head to frequently coughing and constipation.

Bowels opened by a cathartic; nausea and pain in the head continue; p. 90. Haustus, emeticus statim. Diet, tea and gruel.

19. Emetic operated well; feels less pain in the head, p. 90; skin moist, cough less. Mist. salin. antimon. c. tinct. digital. 4ta quaque hora.

P. M. Much better, p. 80. Mist. u. a.

20. Pain in the head with sickness increased this morning, skin moist and cool, p. 80. bowels open. Mist. a. a. Capt. cras primo mane, pulv. ipecac. gr. xviii.

21. Pain in the head continues; emetic operated well; quite free from febrile symptoms; p. 80. App. empl. vesicat. capiti, mistura. u. a.

22. Pain in the head continues without sickness; p. 84. blister rose well. Mist. u. a. empl. vesicat. perpetuum.

23. Pain in the head rather less; p. 90, full; bowels costive. ℞. calomel. gr. v. ext. coloquinth. c. ℈j. M. f. pil. vi. statim sumendae.

P. M. Pain in the head increased; eyes have a glassy appearance; pupils dilated, will contract but little from the light of a candle; intellects deranged; p. 80; skin cool; bowels continue costive. Effluat sanguis ad ℥viii. calomel gr. x. statim; enema stimulans statim et 4ta. quaque hora repetatur si opus erit.

24. As yesterday; pulses rose upon taking away blood to 120, are now 90; not any appearance of inflammation upon the blood. Bowels still costive. Effluat. sanguis ad ℥xii. calomel. gr. v. 4ta quaque hora. ℞. Decoct. commun. ℔j. tinct. sennæ. ℥ß. extr. coloquinth. c. ℈j. natr. vit. ℥ß. M. f. enema, 4ta. quaque hora. Epispasticum majus dorso. Emplastrum perpet. capiti; pedib sinapism.

4. P. M. In every respect as this morning; p. 88. Opened temporal artery, and drew off 12 ounces of arterial blood; pulse began to sink after taking away about 10 ounces; not any marks of inflammation upon the blood taken this morning. Contin. enema stimulans.

8. P. M. Pulse 80, rather feeble; delirium and other appearances of the head as this morning. Bol. Cath. u. a. enem. u. a. The glysters are constantly voided after being retained retained about two hours, with the assistance of an attendant, a little discoloured with fæces.

25. 6 A. M. Pulse 90, rather intermitting; skin quite cool and moist; delirium and appearances of the eyes as before. Sinapisms have occasioned vesication; bowels continue in a state of constipation. ℞. calomel. gr. v. P. antim. gr. iii. ft. bol. 4ta quaque hora um. Haust. seqt. ℞. Tinct. sennæ ʒiii. Infus. sennæ ℥ß. Enem. u. a.

11 A. M. in every respect as this morning.

4 P. M. ditto; p. 80, intermitting.

9 P. M. ditto; p. sinking fast.

26. Died this morning about five o'clock.

I, myself, saw this patient once only on the morning of the 24th; stupor or coma, seemed to alternate with considerable agitation. I was much struck with the glassiness of the eye; the pupil was insensible, the pulse was undulating, and breath cold. Being asked if any plan of practice suggested itself to me, I agreed with others, in considering the case as hopeless, but said, if I were compelled to attempt something, it should be the application of boiling water to the scalp.

Nothing was done, except as above stated.

The dissection took place the evening of the day of death. Little blood, on dividing the integuments. The cranium shewed no vestige of injury. Vessels of the dura mater were turgid, particularly the posterior; at the junction of the sagittal with the lambdoidal suture it was very vascular, thickened, and adherent to the cranium, as also was the falx, and all the membranes to one another, and to the hemispheres of the brain; these and the other parts being a conglutinated mass. The left hemisphere, near the longitudinal sinus, was covered for the area of half a crown with coagulating lymph (fibrine). Vessels of the pia mater by no means turgid; medulla firm, not hard; not marked more, indeed scarcely so much as in representations given for natural, with bloody points; from two to three drachms of lymph in the ventricles; choroid plexus not particularly red or charged with blood, only one turgid vein. There was an appearance, sometimes called an hydatid; it was oval, yellowish, corrugated like the capsules of some fruits, largest diameter not exceeding a goose quill, and three-eighths of an inch long; surface of the corpora striata redder than common. *Tentorium cerebello super extensum* vascular, as if inflamed; vessels below injected. *Thalami nerv. optic.* and the optic nerves free from all appearance of disease. Within the thorax, adhesion hesions between the pleuras; lungs, particularly middle lobe of the left, being tuberculated; tubercles solid, all less than garden peas; membrane of liver, of spleen, and also the peritonæum attached to the diaphragm tuberculated.

This was written immediately on examination. The tubercles *in* the membrane of the spleen were hard and calculous. This viscus being immediately put for 24 hours into an acidulated liquor, I was much surprized at a foam on its surface, arising from the action of slow effervesence; the parts were most perfectly fresh. The calculi (or tubercles) were mostly dissolved out; the cells within the membrane of the spleen being laid open, and just enough to shew the facts remaining of the calculi; not enough, I fear, to ascertain their composition.

Besides Dr. Edgeworth, Mr. Lawrence, and another medical gentleman, who were present at the dissection, the parts have been viewed by Dr. Felix and Mr. Marks of the French prison, and by others. Stomach inflamed, particularly up towards the cardia; the inflamed parts chiefly dark red, with a little florid appearance; portions of the jejunum and ileum inflamed; gangrene had commenced in one spot; intussusception at two places, but these not inflamed.

### CASE V.

A. B. middle aged, well made, accustomed to moderate exertion; said he had been unwell nine or ten days. When he applied for advice, with fever and debility, on the 20th of July, at which time the fever had assumed the typhus character. Pulse 86 in a minute, and small; skin soft, tongue brown, heat moderate, frequently flushed during the night, and perspired very much; bowels regular, appetite bad, thirst not very great.

On the 21st, p. 100 in a minute, tongue brown, moist; skin hot; slept very little in the night; perspired a great deal; says he is perfectly easy; bowels open, thirst moderate.

22d. Slept a little this morning; complains of pain all over his body. P. 120 in a minute, skin hot, tongue brown and dry, teeth furred; diarrhœa came on in the night; feels more thirsty.—22d. 8 P. M. Pulse 130; diarrhœa stopt; in other respects continues as in the morning; dozed a great deal in the day.

23d. P. 100, skin soft, tongue and teeth cleaner; passed a quiet night; slept some; perspired very much; had had two evacuations; mouth sore; has a slight ptyalism; very thirsty.—23d. 8 P. M. Pulse 104, skin hot and dry; sponged with vinegar and water; he has had two motions during the day.

24th. Passed a very good night, and in most respects as yesterday.—24th. 9 P. M. Pulse 130, small; tongue moist; dozes and perspires a great deal; feels perfectly easy, and less thirsty.

25th. P. 120, skin soft, tongue moist; slept tolerably in the night; bowels open.—8 P. M. Continues as in the morning; p. 128.

26th. P. very quick and small; has been rather delirious at times in the night; had two motions since the evening; skin soft, hands feel cold; every thing taken is vomited up.—26th. 8 P. M. Pulse 140, small, skin hot and dry, tongue furred; since 10 o'clock this morning what he took has remained on his stomach.

27th. He appears much weaker; p. exceedingly quick and small; other symptoms as yesterday; is now perfectly sensible.—8 P. M. Continues as in the morning.

29th. Pulse feeble and quick, skin soft, tongue much furred; passed a restless night, was delirious; extremities cold, bowels open. He died at six o'clock in the evening.

The general treatment, I was informed, besides the sponging, as mentioned, consisted of antimonials, opiates, acids, and wine, in the last days. I was particularly told that he had never appeared hot enough for affusion, according to Dr. Currie's rules. I never saw this patient alive, but examined another in the same room, said to be affected in the same manner, except that he had great and steady heats, and the affusion was used. This recovered very slowly.

July 30. Body opened at noon, perfectly fresh; no froth, effusion, or remarkable external change; hardly any livid patches (death spots) on the depending parts of the skin. While the cranium was most carefully sawing through, effusion took place, first of bloody serum, afterwards of blood from the left ear, not one drop having previously flowed. The cranium being neatly separated, this effusion was ascertained to come from the *meatus extern. audit.* It was absorbed by the linen. We (two surgeons and myself) estimated it at 2 oz. The dura mater exhibited preternatural vascularity; the arterial trunks being wide in the diameter; their centre occupied by a crimson coagulum; the sides looking white and tendinous; an appearance common common after hydrocephalus internus, and other inflammations, but not peculiar to these; only the appearance seems favourable for judging of the size of the arteries; longitudinal and lateral sinuses not turgid; veins in the sulci of the brain rather full; arteries every where ramifying over the eminencies, and finely injected. Brain fresh, firm, not hard. On slicing the left hemisphere, innumerable bloody points were seen in the medulla, and also less numerously and strikingly on the cut cortical part: from these points, on making a parallel cut with a broad thin knife, blood very freely issued; lateral ventricles scarcely contained a drachm of liquid; vessels on the *corpora striata* turgid; choroid plexus a dark bloody mass, six times its average size; its vessels all turgid; arteries visible as crimson streaks upon the optic nerve, fro the cut sub-m stance of which oozed spots of blood in several points, between the centre and the circumference. Pia mater under the anterior part of the brain excessively injected. The dura mater on the hollow of the occipital bone much more vascular than any where else. If from gravitation after death?

The cerebellum, medulla oblongata and spinalis, offered the same appearances of injection externally, and bloody points internally.

Thoracic muscles firm, ruddy, fresh; surface of the lungs offered a white ground beautifully diversified with dark specks. They were spungy and healthy, except the back of the superior lobe on the right side, which was denser, and more liver-like than could well arise from gravitation of the blood in the time. The membrane lining the trachea was of a deep red, and appeared highly inflamed, so that the most successful injection could scarce attain to colour it so deeply and so uniformly. This extended into the ramifications. [The appearance led me to enquire, if there had been any cough during the illness; there had not been the least, nor any dyspnœa but what might arise from debility.] Heart small, firm, natural. Cavity of abdomen natural. Omentum without fat, excessively injected. Strokes with a fine pen and crimson ink, in some places as close as is consistent with distinctness, in others far broader and apart, the paper representing the transparent membrane, would give an idea of the appearance; spleen thrice the natural size, otherwise seemingly healthy; pancreas hard, liver rather pale; from the gall bladder, which was ruddy and streaky, not the least transudation of bile, nor could it be guessed what coloured liquid liquid it contained. It was moderately full, and being punctured apart, discharged a yellow liquid, charged with mucous flocculi.

Another circumstance was discovered in this patient, exhibiting, if I mistake not, the first rudiments of a curious bilious affection, not described in any of our late treatises, yet in general easy to be known, and when known, accounting for certain appearances in disease, and directing treatment. I shall some time explain it, if no one anticipate me.

Stomach with the rugae firm, and well preserved; villous coat intire, pale, and free from inflammation every where towards the large curvature. Towards the cardia a patch chiefly of the most highly florid inflammation, of the extent of a crown piece. It continued less intense into the lower part of the œsophagus, not high up. Intestines every where shewing patches considerably inflamed, particularly the colon: a small tract of the jejunum was emphysematous, the air beneath the peritoneal coat; adhesions between the cœcum and colon; kidneys surprisingly flabby for so fresh and firm a subject, not diseased within. The notes of this examination were written on the spot, as the appearances presented. The same attention was paid to accuracy in some other instances, here related; and when the account was deferred till I had reached home, it was always drawn up immediately, and now and then confronted with statements by other persons present. Only on one occasion were the appearances surveyed by two pair of eyes; in general, three or four people were present.

The absence of cough, &c. in this case is a striking fact, and the vomiting on the 25th, independent of medicine, with its cessation on the 26th, is another.

The two gentlemen present, with myself, repaired immediately from this inspection to the patient, whom I have mentioned, as similarly affected. We roused him from a slight delirious stupor; his faculties were then distinct. I pressed on various parts, and he said he felt no pain; but on doing the same, as well on the trachea as on the pit of the stomach, with intermediate removal of the finger elsewhere, he acknowledged pain in both those places, and only those; and I believe, in fever, it will be of importance to prosecute this method of search more minutely than has ever been the practice. Clearly detected soreness should encourage bleeding at an early stage.

### CASE VI.

This patient I did not see alive. W. J. aged 24, accustomed to moderate exertion, and temperate; complains, August 29th, of very great debility, and states, that for three days previous he had, on an average, from fifteen to twenty motions in the twenty-four hours, accompanied with incessant vomiting, a continued pain in his stomach; and he then described himself, as feeling excessively cold, although his skin was hot and dry, and pulse 100. Capt. haust emet: statim. post operat: solution: salis.

Evening. The emetic and the aperient operated very favourably, and he feels himself considerably relieved. Tongue furred, skin feverish, pulse full, and 80. Haust. salin. anod. with t. opii gtt. xx.

30th. Had a tolerable night, and three very loose motions; skin warm, pulse 74, heat moderate. Considers himself much better. Solution: antimon. tartar. gr. ii. in aq. ℥vi. cochl. ii. omni horâ.

Evening. Little alteration. Haust anod.

31st. Had a very restless night; stools frequent and dark, pulse 100; tongue foul, skin hot and dry. Consider the antimony not to have had time to excite a diaphoresis. Cont. solutio antim. tartar.

Evening. Skin moist, pulse 84. The stools less frequent and of a better colour. Haust. salin. anod.

Sept. 1st. Tongue very foul, stools involuntary, p. 100; pain in his head; has had a very restless night, occasionally delirious. Emp. vesicat. capiti, pulv. ipecac. comp. ℈ß. sextis horis.

One o'clock P. M. No appearance of approaching diaphoresis. On taking the Dover's powder, he instantly threw it up, accompanied with a quantity of green matter; the disease now synochus soporosa. ℞. Pulv. ipecac. gr. xv. statim. wine two gills.

Half-past three. P. M. The emetic operated mildly; he has had one motion of a more favorable consistence and colour. ℞. Mist. camphorat. ℥vß. tinct. opii gtt. xl. vin. antim. ʒi. æther. vitriolic. ʒiß. cochl. ii. omni hori.

Thirty-five minutes past eight, P. M. The patient has been extremely delirious; about five o'clock he, however, became more collected; had two motions of much better appearance, but very loose; pulse 80; tongue very foul and very black; great predisposition to vomit. Wine two gills. Haust. camphorat. anod. statim. emp. vesicalorium inter inter scapulas; yeast two spoonfuls every hour, omitting every other medicine.

Sept. 2d, morning visit. The yeast, after the first dose, was repugnant to him; no means or persuasion could get him to repeat it; he has had but one stool during the night and that very dark, and very offensive, yet not so much so as those of yesterday; he has just had however, two more motions, nearly of the same description, *immediately* succeeding each other; occasionally delirious, but the intervals of sanity much longer. The blister between his shoulder rose extremely well, whereas that applied last night, has at present no appearance of effect; skin warm, pulse 100; tongue very black, with a black incrustation, and very dry. An attempt to get down some cordial mixture has failed, as well as a succeeding one to get down some simple saline mixture, in a state of effervescence; fancies he could take pills. ℞. Ext. opii gr. j. pulv. camphor. gr. x. fiant pilulæ statim v. sumendæ, et rep. tertia quaque hora; a pint of sherry and lemons.

Two o'clock, P. M. He could not take the pills, as in swallowing one, the vomiting of the green matter returned in a greater degree than before; no stool since morning visit. Pulse and tongue, as before. ℞. Antim. tartar. gr. ii. aq. puræ ℥ij. capt. ℥ß. statim et rep. o. horae dimid. donec vomitum cierit.

Evening visit. No alteration since the morning, except that since the emetic the vomiting has ceased, and he keeps the wine on his stomach, the only thing he can now take; the debility is extreme. Habt. tinct opii gr. xl. h. s. in his wine.

Sept. 3d. Vomiting returned; two motions as before described since last night. Every other symptom much worse; he now refuses the wine. Brandy half a pint with lemons. Spt. æther. gtt. xx. tertia quaque hora. Evening, a-sleep.

Nine o'clock, P. M. Symptoms the same, but much worse; no motion. Enema comm. statim.

4th Morning. Wholly insensible; repeat the brandy and lemons; died at half-past 3 in the afternoon. Body opened between 7 and 8 in the morning of the 4th. On the back some death marks; not a drop of blood from the cut integuments of the cranium, nor did any ooze from the divided temporal or occipital muscles; the scalpel was scarce stained red. After dissection of the head, only a slight patch of blood on the block which supported it. Vessels of the dura mater in a medium state, less injected than in

Mr. Charles Bell's 1st plate, which I carried with me for comparison; the veins in the sulci less turgid, and the cut medullary substance, in several sections, shewed far fewer bloody points than he has exhibited. Substance of the brain firm, corpora striata slightly vascular; choroid plexus by no means deep red, turgid, or large; two or three drachms of lymph in the ventricles; nothing different or remarkable in the cerebellum.

Thoracic muscles firm, of a fine red; no vestage of putridity; lungs, liver, gall-bladder, healthy; no transudation of bile, gall-bladder detached, and a table spoonful of dark green bile received pure into a clean phial. The stomach was full of a strong smelling darkish brown fluid, of which some was secured in a phial: villous coat no where abraded or dissolved, but every where covered with bloody dark brown mucous matter. This being wiped off, a florid inflammation was seen every where dispersed in patches, but particularly high and thick about the cardia. There appeared intense, red spots, as if ready to pour out arterial blood; nothing verging to grangrene. The intestines were inflamed in spots, and every where full of the same offensive liquid as the stomach.

This copious liquid appeared to consist of blood, bile, (it differed altogether from the contents of the gall-bladder) of mucus, and other secretions. Its smell was somewhat alliaceous, clearly distinguishable from putrid, nor did it tarnish bright silver. The medical gentleman, who performed the dissection principally, had been accustomed to open bodies after the yellow fever, and observed that it differed in scent and colour from black vomit, which nearly resembles coffee grounds. This was confirmed by Dr. M'Gregor, who has watched the sick and prosecuted dissections in so many climates. I shewed the liquor to him before it was sensibly altered. In some future observations on animal fluids I may enter farther into an account of it. That it was mostly secreted by the stomach and bowels, was probable; but another case had already afforded me clear proof of this fact, as I shall state hereafter.

Within a few months, I have been present at the opening of four bodies, where death was certainly instantaneous in two, probably so in a third, and such as might be called sudden in the fourth.

### CASE VII.

An intemperate middle-aged blacksmith, had been last winter confined some weeks by a complaint denominated asthma.

asthma. Thinking he should soon be able to return to his workshop, he walked up to one of his house windows, from which it was visible; and at the window dropped down and expired without a groan. A medical gentleman, called in haste, attempted to bleed him. Next morning (I believe) he was opened. Some blood having flowed from one ear, it was confidently expected that the head would exhibit much congestion or extravasation; but the membranes had their vessels neither turgid nor injected. The surface of the corpora striata was faintly vascular, the choroid plexus not large, or full of blood. There was scarce a bloody point in the medulla. The substance of the brain was exceedingly soft and easy to tear. In the chest was nothing but a small solid tubercle, which, perhaps, never excited any symptom. The stomach and abdominal viscera were free from any apparently fatal change. An hydatid, the size of a nut, was found rising to the surface of the kidney.

Instances like this are familiar. They abound in the papers belonging to foreign medical jurisprudence, yet it requires ocular demonstration to place implicit confidence in apoplexy, the opposite to sanguineous; and the disappointment will be greater, where external effusion of blood takes place. But the opposite results of inspection are strikingly analogous in apoplexy, and in typhus or other fever.

### CASE VIII.

A young woman, dark and robust, was early subject to startings in her sleep, and to frightful dreams, from which she awaked for sometime delirious. From her 8th to her 19th year, these symptoms were not complained of. Then a domestic vexation was followed by a fit, from description epileptic, or certainly violently hysterical. Such fits recurred, especially from chagrin, at no distant intervals. She must have been aware of their approach, as she was said to lock herself up alone before they came on. She had engaged in an active business though single, and had given imprudent credit. The day of her death, she wrote a letter to one of her debtors: and the prospect of loss probably preyed on her irritable mind.

July 4th 1808. (Being then about 23 years of age) she went from her lodgings to breakfast in her shop, at two or three hundred yards distance. Here she eat a little and drank tea, and about 9 A. M. seemed to a relation's ser-

 vant vant very low spirited. She was at first pale, afterwards flushed, complaining so much of her head, and of her head only, that the girl brought her a pillow, against which to lean it. At 11 an acquaintance found her in the shop, complaining also greatly of head-ach, and saying she had not long to live. About this time she had begun a letter, which I saw, to her mother, repeating, in the melancholy prolixity of desponding affection, that she should live but a few hours, and that she hoped they should meet in a happier world. Her acquaintance returning by noon, found the shop door fastened. When it was forced open, the patient was found senseless in a fit. A medical gentleman found her about one "in every respect," to use his own words, "as is usual in common cases of fits." He left her expecting she would recover. About three P. M. she did recover enough to recognize a child, her relation, stroking her arm, and uttering a few words collectedly, but immediately relapsed. Another medical gentleman ordered her a table spoonful of a solution of x grains of tartar emetic in 6 oz. of water every half hour, of which it is believed four doses were forced down, but without effect. At four the first attendant found her foaming at the mouth with a bloated countenance, lividness of the lips, face, and hands, appearance of suffocation, full pulse, and damp skin. This was nearly her state at first. He divided the temporal artery. This yielding scarcely any blood, he took eight oz. from the arm. After nine P. M. I found her with open mouth, stertorous respiration, black tongue, p. at the wrist 86, full; carotid and temporal arteries beating moderately; pupil much contracted, insensible, skin clammy; a quarter before ten o'clock she died.

At 7 A. M. next morning, she was opened; Mr. King, surgeon, Hotwells, Mr. Rolfe of Bristol, and two medical pupils being also present.—Abdomen had swelled during the night; much foam had issued continually, as the nurse said, and was actually issuing when we assembled, from the nostrils; ten hours after death.

The scalp was divided some time before the cranium was sawed through; large drops of dark, speedily coagulating, blood issued on the first incision, and in half an hour, (during which the other cavities were opened) it amounted to perhaps an ounce. Vessels of the dura mater large, and unusually injected; veins of the p. mater in the furrows turgid; crimson vessels finely ramified, in vast numbers, upon the eminencies of the brain, exceeding, perhaps, Mr. Ch. Bell's highly coloured pictures. Substance of the brain brain remarkably soft and flaccid; wherever the medulla was divided, blood started from crouded points, and continued to ooze on slight pressure; little lymph in the ventricles; many vessels ramifying on the corpora striata; scarce any vestiges were left of the corpus lucidum; (I have seen this absorbed in hydrocephalus); cerebellum amazingly blood-shot, its substance soft and spungy; blood, as before, started from every point where it was cut.

Lungs very healthy; coronary vessels and left ventricle of the heart turgid with blood; in the abdomen was a slight red appearance on the surface of the intestines, particularly those depending; colon contracted in places; stomach externally white, and its substance thick, containing a good deal of air, with four ounces of a mucous and bilious fluid (which being examined by the senses and chemical tests, was found to contain no suspicious ingredient). The rugae of the stomach prominent; its interior extensively, highly, and floridly inflamed, particularly about the cardia. Besides the florid redness, one patch, less than half-a-crown, was livid, and as one of the spectators thought, abraded. I could only perceive more spunginess of the villous coat than elsewhere.

In the ovaria were hydatids; in the Fallopian tubes, an indistinct praeternatural formation; in the uterus a spungy vascular substance, difficult to describe, but totally different from any product of conception. An oval ball of thread with blood vessels intertwisted, and lining the sides, will give some gross notion of the thing. It shewed nothing membranous like the coats of the ovum; vagina large; little appearance of hymen.

It was impossible to contemplate a case, like this, for five minutes without certain suspicions, particularly as the patient had written a prediction of her death within a few hours. Upon weighing all circumstances, my own suspicions totally vanished, and I believe those of the other medical persons. The sense of imminent dissolution belongs to nervous affections, and I suppose that this fact goes to prove, that an ingenious distinction between hysteria and epilepsy does not hold good; or was this hysteria ending in apoplexy? A girl of good character afterwards attested to me, that this patient ceased to menstruate only on Saturday night, the 25th of June, nine days before her death. "She *shifted herself on Sunday morning*," was the expression of this girl, her bed-fellow.

The existence of such intense inflammation in both the stomach and head is remarkable It appears as certain as any any thing can be, respecting internal movements, that the head was here first affected, since she eat and drank after the head-ache became very severe, and did not at all complain of her stomach—which perhaps is less conclusive, as nobody pressed it till I came, and then sensation had long been abolished. Some facts which I have quoted from De Haen in my *Researches on Fever,* coincide most strikingly with this *projection of inflammation* from the head. I shall not now enlarge upon the topic.

I have met with evidence enough of inflammation wandering from external injuries far over the system.

The following fact is the only one which I shall give at present

A large mongrel bred spaniel was struck by a thrown stone, on the right side of the loins. A few days afterwards, being tapped or patted on the spot, he howled prodigiously, as if in most violent pain; he thenceforth began to droop, hung his tongue, yet eat and lapped a little; contrary to his habits, he grew fierce towards his own species, sallied forth to attack all that passed; bit a cat of the house, an old favourite of his own; never tried to bite any of the family; died in a fortnight. This I learned. August 4th, after being dead two nights and a day it was opened. The delay was unlucky, but the distinction between the condition of parts, even in the same organ, was still striking; kidneys gangrenous; the right, over which the blow had been received, most highly so, almost a fluid mass of putrefaction; stomach and intestines highly inflamed *in patches*; liver inflamed; trachea of a brown red, with uniform intense inflammation; upper part of the œsophagus the same, but it was pale and natural below, nor were the bronchial glands or the salivary inflamed. Vessels of the membranes of the brain much injected.

### CASE VIII.

A full, bloated, above middle-aged, and most intemperate drinker, who might be called *a moving fixture* of this place, after a dinner of pleasure, was brought home dead. Examination on oath could draw forth nothing clear as to the manner of his death. It had probably been immediate, and he had fallen. It certainly happened in the evening, within less time than an hour and half, while travelling in a carriage. In the morning he had felt uncommonly well. Some time after death, the jugular was pierced; at the time and after, it was stated that a quart of blood had issued issued. It was the middle of August, and forty hours had elapsed before consent could be obtained to open the body, which however lay in a shaded N. W. room, and was not nearly so putrid as might be expected. There was nothing indeed very offensive till the abdomen was opened. A very diffused black, or livid colour over the left side of the face (as if from a blow or fall); left temporal muscle more tender and discoloured. Scalp and cranium thick. Dura mater as closely agglutinated to the cranium as paper hangings to a wall. If the subject had been trepanned, the dura mater must have been cut through, and could not have been detached, if it had not. (This agglutination, doubtless, from chronic inflammation). *Pia mater* much thickened;—much injected, particularly on the left side, where the bruise was. At the summit of the hemispheres, some effusion of coagulating lymph; substance of the brain very soft; many large bloody points in the cut medulla; about two drachms of bloody lymph in the *left* lateral ventricle, (towards which side the head, during dissection, also inclined) choroid plexus turgid; great redness about the cerebellum; coats of the optic nerves much streaked with blood vessels; lateral sinuses full of blood, and the dura mater descending into the vertebral sheath much injected. Adhesions between the left pleuras; pleura pulmonalis very thick; (a natural structure? the patient had laboured under pneumonic affections;) liver hard, nearly of the colour of some blue clays; stomach and intestines appeared healthy, having undergone little change since death.

I have used and quoted Mr. Charles Bell's engravings, merely because they are much in the hands of medical men. I do not question that he has exhibited what he saw in the objects from which he drew; but I am persuaded that his colouring deviates as widely as an enamelled face from nature. Whoever sees a pia mater equally vascular with his representation may safely swear to great inflammation. This has been assented to by several actual observers. One found by measurement that the diameter of the blood-vessels, as given by Mr. Bell for natural, is greater on his reduced scale, than in some degree of inflammation. Those plates possibly represent vessels, first distended by disease, and afterwards by injection. I shall produce further facts and observations on this subject hereafter.

I conclude for the present with a philological, perhaps, an unimportant remark. In conformity with a custom, originating I believe with a physician in London of vast reputed skill, derived by him probably from an ill-contrived trived phrase of Mr. Hunter, and beginning to grow inveterate,* I have formerly called that branch of medical knowledge, to which the above facts belong, *morbid anatomy.* I felt some compunction of conscience at the moment; and I now openly beg pardon of the genius of good taste for my offence. If in medical writings, we are to pay any attention whatever to propriety of language, we must forthwith abandon this clumsy morsel of jargon!

I should, indeed, be glad to find any scholar trying to translate "*the morbid anatomy of some of the most important parts of the human body,*" closely into any civilized dialect; Greek, Latin, French, or English.

Ανάτομη νοσουσα.

Anatomia ægrotans.

Anatomie maladive.

Diseased anatomy.

The dextrous hand of Dr. Soemmering could not, I see, mould his stuff at will; so for *krankhafte Zergliederungskunst,* he was obliged to shove in *krankhafter Bau.*

The truly eminent French enquirers write *anatomie pathologique.* One cannot object to the title of Morgagni without being *morbidly* censorious. Nothing can be worse than the term of our own countryman. And for my part, I can find nothing better than *pathological anatomy.* For although I much prefer simple short old English words to long Greek compounds, yet pathological, or that which accounts for disorders, is unquestionably expressive; in *physiological* we have a precedent most strikingly apposite; and I trust we shall all rather submit to two hellenisms than one barbarism.

This is a trifling criticism, and the question seems scarcely to admit of dispute. The merit of the work, to which so objectionable a title stands prefixed, is a deeper and more important question. I have been led long since to consider it seriously; and in the sequel I shall take the liberty of inquiring, whether the authority of the writer ought to be held much more sacred for the thing, than for the word.

*Clifton, Sept.* 19, 1808.

* Thus, e. g. we are just favoured with *Essays on the morbid Anatomy of the Human Eye.* They might with the same propriety have been entitled, *Morbid Essays on the Anatomy of the Human Eye.*

Cases

